# Azacitidine for Front-Line Therapy of Patients with AML: Reproducible Efficacy Established by Direct Comparison of International Phase 3 Trial Data with Registry Data from the Austrian Azacitidine Registry of the AGMT Study Group

**DOI:** 10.3390/ijms18020415

**Published:** 2017-02-15

**Authors:** Lisa Pleyer, Hartmut Döhner, Hervé Dombret, John F. Seymour, Andre C. Schuh, CL Beach, Arlene S. Swern, Sonja Burgstaller, Reinhard Stauder, Michael Girschikofsky, Heinz Sill, Konstantin Schlick, Josef Thaler, Britta Halter, Sigrid Machherndl Spandl, Armin Zebisch, Angelika Pichler, Michael Pfeilstöcker, Eva M. Autzinger, Alois Lang, Klaus Geissler, Daniela Voskova, Wolfgang R. Sperr, Sabine Hojas, Inga M. Rogulj, Johannes Andel, Richard Greil

**Affiliations:** 1 3rd Medical Department with Hematology and Medical Oncology, Hemostaseology, Rheumatology and Infectious Diseases, Laboratory for Immunological and Molecular Cancer Research, Oncologic Center, Paracelsus Medical University, Salzburg 5020, Austria; k.schlick@salk.at (K.S.); r.greil@mac.com (R.G.); 2 Salzburg Cancer Research Institute—Center for Clinical Cancer and Immunology Trials, Salzburg 5020, Austria; 3 Cancer Cluster, Salzburg 5020, Austria; 4 Department of Medicine and Internal Medicine III, Universitätsklinikum Ulm, Ulm D-89081, Germany; hartmut.doehner@uniklinik-ulm.de; 5 Institut Universitaire d’Hématologie, Hôpital Saint Louis, University Paris Diderot, Paris 75010, France; herve.dombret@mac.com; 6 Peter MacCallum Cancer Centre, East Melbourne, Australia, and University of Melbourne, Parkville 3000, Australia; john.seymour@petermac.org; 7 Princess Margaret Cancer Centre, Toronto, ON M5G 2M9, Canada; andre.schuh@uhn.ca; 8 Celgene Corporation, Summit, NJ 07901, USA; clbeach@celgene.com (C.L.B.), aswern@celgene.com (A.S.S.); 9 Department of Internal Medicine IV, Klinikum WelsGrieskirchen, Wels 4600, Austria; sonja.burgstaller@klinikum-wegr.at (S.B.), josef.thaler@klinikum-wegr.at (J.T.); 10 Department of Internal Medicine V, Innsbruck Medical University, Innsbruck 6020, Austria; reinhard.stauder@i-med.ac.at (R.S.), britta.halter@i-med.ac.at (B.H.); 11 Department of Hematology and Oncology, Elisabethinen Hospital, Linz 4020, Austria; michael.girschikofsky@elisabethinen.or.at (M.G.), sigrid.machherndl-spandl@elisabethinen.or.at (S.M.S.); 12 Department of Hematology, Medical University of Graz, Graz 8036, Austria; heinz.sill@medunigraz.at (H.S.), armin.zebisch@medunigraz.at (A.Z.); 13 Department for Hematology and Oncology, LKH Leoben, Leoben 8700, Austria; angelika.pichler@kages.at; 14 3rd Medical Department for Hematology and Oncology, Hanusch Hospital, Vienna 1140, Austria; m.pfeilstoecker@aon.at; 15 First Medical Department, Center for Oncology, Hematology and Palliative Care, Wilhelminenspital, Vienna 1160, Austria; eva-maria.autzinger@wienkav.at; 16 Department of Internal Medicine, Landeskrankenhaus Feldkirch (LKH) Feldkirch, Feldkirch 6800, Austria; alois.lang@lkhf.at; 17 5th Medical Department, Hospital Hietzing, Vienna 1130, Austria; klaus.geissler@wienkav.at; 18 Department of Internal Medicine III, General Hospital, Linz 4020, Austria; daniela.voskova@gmail.com; 19 Department of Internal Medicine I, Division of Hematology and Hemostaseology, Medical University of Vienna, Vienna 1090, Austria; wolfgang.r.sperr@meduniwien.ac.at; 20 Department of Internal Medicine, LKH Fürstenfeld, Fürstenfeld 8280, Austria; sabineelisabeth.hojas@kages.at; 21 Department of Hematology, Clinical Hospital Merkur, Zagreb 10000, Croatia; imandac@yahoo.com; 22 Department of Internal Medicine II, LKH Steyr, Steyr 4400, Austria; johannes.andel@gespag.at

**Keywords:** acute myeloid leukaemia (AML), AZA-AML-001 trial, Austrian Azacitidine Registry (AAR), real-world data, azacitidine

## Abstract

We recently published a clinically-meaningful improvement in median overall survival (OS) for patients with acute myeloid leukaemia (AML), >30% bone marrow (BM) blasts and white blood cell (WBC) count ≤15 G/L, treated with front-line azacitidine versus conventional care regimens within a phase 3 clinical trial (AZA-AML-001; NCT01074047; registered: February 2010). As results obtained in clinical trials are facing increased pressure to be confirmed by real-world data, we aimed to test whether data obtained in the AZA-AML-001 trial accurately represent observations made in routine clinical practice by analysing additional AML patients treated with azacitidine front-line within the Austrian Azacitidine Registry (AAR; NCT01595295; registered: May 2012) and directly comparing patient-level data of both cohorts. We assessed the efficacy of front-line azacitidine in a total of 407 patients with newly-diagnosed AML. Firstly, we compared data from AML patients with WBC ≤ 15 G/L and >30% BM blasts included within the AZA-AML-001 trial treated with azacitidine (“AML-001” cohort; *n* = 214) with AAR patients meeting the same inclusion criteria (“AAR (001-like)” cohort; *n* = 95). The current analysis thus represents a new sub-analysis of the AML-001 trial, which is directly compared with a new sub-analysis of the AAR. Baseline characteristics, azacitidine application, response rates and OS were comparable between all patient cohorts within the trial or registry setting. Median OS was 9.9 versus 10.8 months (*p* = 0.616) for “AML-001” versus “AAR (001-like)” cohorts, respectively. Secondly, we pooled data from both cohorts (*n* = 309) and assessed the outcome. Median OS of the pooled cohorts was 10.3 (95% confidence interval: 8.7, 12.6) months, and the one-year survival rate was 45.8%. Thirdly, we compared data from AAR patients meeting AZA-AML-001 trial inclusion criteria (*n* = 95) versus all AAR patients with World Health Organization (WHO)-defined AML (“AAR (WHO-AML)” cohort; *n* = 193). Within the registry population, median OS for AAR patients meeting trial inclusion criteria versus all WHO-AML patients was 10.8 versus 11.8 months (*p* = 0.599), respectively. We thus tested and confirmed the efficacy of azacitidine as a front-line agent in patients with AML, >30% BM blasts and WBC ≤ 15 G/L in a routine clinical practice setting. We further show that the efficacy of azacitidine does not appear to be limited to AML patients who meet stringent clinical trial inclusion criteria, but instead appears efficacious as front-line treatment in all patients with WHO-AML.

## 1. Introduction

Acute myeloid leukaemia (AML) is predominantly a disease of the elderly, with a median age at diagnosis of roughly 70 years [1]. Older patients with AML are often precluded from intensive treatment, owing to multiple poor-risk prognostic factors, including a higher proportion of adverse cytogenetics, myelodysplasia-related changes (MRC), poor Eastern Cooperative Oncology Group performance status (ECOG-PS) and/or significant comorbidities [1,2,3,4,5]. In 2008, azacitidine was approved by the European Medicines Agency (EMA) for the treatment of AML patients with 20%–30% bone marrow (BM) blasts, older than 64 years and who are ineligible for HSCT. For AML patients with more than 30% BM blasts, azacitidine remained an off-label indication until 30 October 2015 and was not reimbursed in most countries. These patients were either treated with other options or not treated at all, which is substantiated by a large population-based study published in 2012, showing that only 38% of 5480 AML patients older than 65 years received leukaemia therapy, whereas 62% received BSC [6]. In Austria, off-label drug use is permitted if a dire clinical need, lack of alternative substances and presumed efficacy can be substantiated. Haematologists at specialized centres started treating AML patients with >30% BM blasts with azacitidine as early as 2007 in Austria, indicating that the physicians were convinced they were doing the best for their patients. This assumption was based on the significant improvement of overall survival (OS) obtained in the AZA-MDS-001 trial [7] and the Cancer and Leukemia Group B (CALGB) protocols, in which 32% [8] and 38% [9,10] of the trial population had AML with 20%–30% BM blasts, respectively. In anticipation of a need to test and potentially confirm the efficacy of azacitidine in a real-world population, the Austrian Azacitidine Registry (AAR) of the Arbeitsgemeinschaft Medikamentöse Tumortherapie (AGMT) Study Group was founded in February 2009 to establish a platform to document the off-label use of azacitidine [11,12,13,14,15,16,17,18,19,20].

In 2010, the international phase 3 randomised AZA-AML-001 clinical trial testing azacitidine versus conventional care regimens (intensive chemotherapy, low-dose cytarabine or BSC as preselected by the treating physician) in AML patients older than 65 years with newly-diagnosed AML, >30% BM blasts and ≤15 G/L white blood cell (WBC) count was initiated. In this trial, a clinically-meaningful improvement in OS for azacitidine versus conventional care regimens (10.4 vs. 6.5 months; *p* = 0.1009) was reported by part of our group on 16 July 2015 [21], and EMA approval of azacitidine was expanded on 30 October 2015 to include AML patients with >30% BM blasts.

While well-designed randomised clinical trials are the gold-standard for investigating treatment options for (AML) patients, they are considered to include extremely selected, skewed and/or not fully-representative patient populations [22]. Results obtained may therefore under-represent some patient groups [22], for example those with comorbidities or anticipated intolerance to treatment [23]. Thus, caution needs to be exercised when using newly-approved drugs in patients who would not have met clinical trial inclusion criteria, and conclusions drawn from clinical trials cannot eo ipso be generalized to all patients with AML. In fact, experts in the field have recently ascertained that all trial results should be extrapolated with caution, and population-based studies of real-world patients have a prominent role in examining the prognosis, as well as the management, efficacy and toxicity of new agents after regulatory approval and outside of clinical trials of higher-risk MDS [24,25] and AML [26].

**Figure 1:**
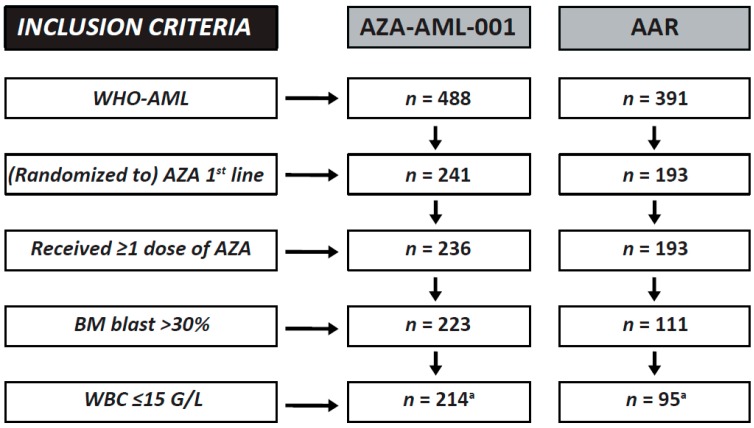
CONSORT diagram. ^a^ Subset of patients included in pooled analysis (AML-001-like).

Therefore, a cooperative data analysis between the sponsors and data owners of the AZA-AML-001 trial and the AGMT-AAR-registry was agreed upon, with the aim to test whether the use of azacitidine within the routine clinical practice setting can recapitulate the median OS time of 10.4 months observed in AML patients with >30% BM blasts and ≤15 G/L treated with azacitidine front line within the AZA-AML-001 trial. Of note, 27 of the 241 patients (11.2%) treated with azacitidine within the AZA-AML-001 trial were excluded from the current analysis, as they had ≤30% BM blasts after central pathology review and, therefore, did not fulfil the inclusion criteria of this study (Figure 1). The current analysis thus represents a new sub-analysis of the AML-001 trial, which is directly compared with a new sub-analysis of the AAR. Secondly, we wanted to expand on the data from the AZA-AML-001 trial regarding the efficacy of azacitidine as front-line therapy for AML patients [21,27,28], by analysing additional patients from the AAR and directly comparing the patient-level data of both cohorts. Thirdly, we aimed to analyse whether the efficacy and safety of azacitidine as a front-line agent in AML is limited to patients meeting strict clinical trial inclusion criteria by analysing and directly comparing patient-level data from a wider patient population of the AAR that includes all patients with AML as defined by the World Health Organization (WHO).

## 2. Results

### 2.1. Clinical Trial versus Registry Subsets

Our predefined criteria (>30% BM blasts and ≤15 G/L WBC) for the direct comparative analysis of the clinical trial with registry data were met by 214 patients of the AZA-AML-001 trial (“AML-001” subset) and 95 patients from the AAR (“AAR (001-like)” subset) (Figure 1). This represents a novel and thus far unpublished subset of the AML-001-trial, as 27 of the previously published cohort of 241 patients [21] were excluded, as they had <30% BM blasts after central pathology review.

Baseline characteristics were similar between the “AML-001” subset and the “AAR (001-like)” subset, including gender, median age, median BM blast count, WBC count, haemoglobin, absolute neutrophil count (ANC), platelets (PLT), prior myelodysplastic syndromes (MDS), transfusion dependence (TD) and National Comprehensive Cancer Network (NCCN) cytogenetic risk (Table 1). The only notable differences were the presence of patients with ECOG-PS 3–4 (9.5%; nine patients) and the presence of patients with good-risk cytogenetics (2.1%; two patients) within the “AAR (001-like)” subset, both of which were exclusion criteria in the “AML-001” subset (Table 1).

Treatment-related characteristics, such as median number of azacitidine cycles (six vs. five), percentage of patients receiving ≥6 cycles (50% vs. 46%) or ≥12 cycles of azacitidine (29% vs. 24%), median total days of azacitidine application (42 vs. 34 days), daily dose (130 vs. 132 mg), patient status at data cut-off and reasons for azacitidine discontinuation were similar between the clinical trial and registry cohorts, respectively (Table 2).

**Figure 2:**
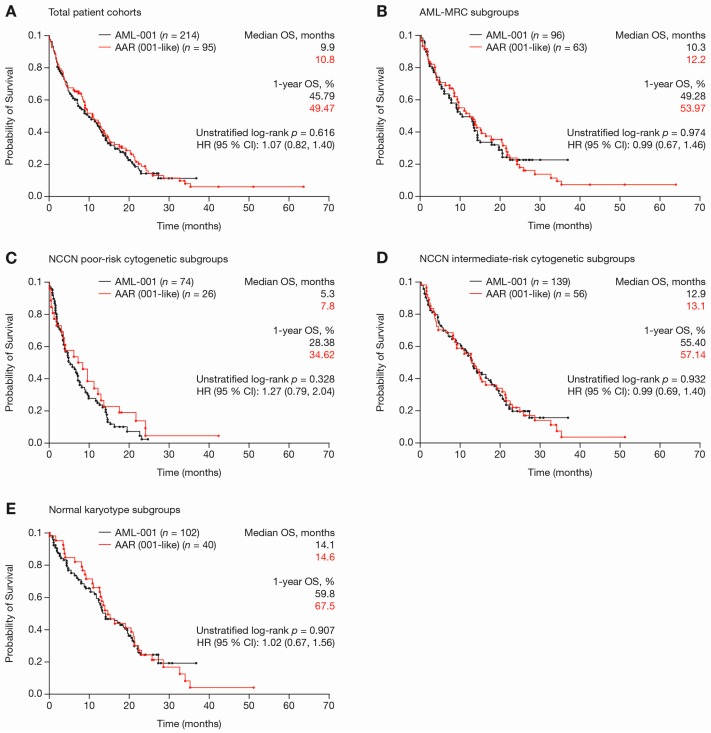
Overall survival (OS) in AML patients with >30% BM blasts and <15 G/L WBC treated with azacitidine front-line within the AML-001 trial and the AAR-AML-001-like cohorts. (**A**) Total patient cohorts; (**B**) AML-MRC; (**C**) AML with NCCN poor-risk cytogenetics; (**D**) AML with NCCN intermediate-risk cytogenetics; and (**E**) Normal karyotype.

Median OS (9.9 vs. 10.7 months, *p* = 0.9553), median relapse-free survival (RFS; 16.3 vs. 13.8 months, *p* = 0.6817), median event-free survival (EFS; 6.9 vs. 8.3 months, *p* = 0.2909), median complete response (CR)/CR with incomplete blood count recovery (CRi) duration (8.6 vs. 11.1 months, *p* = 0.1740), one-year survival rates (54.2% vs. 53.7%, *p* = 0.924) and 30-day mortality rates (7.0% vs. 8.4%, *p* = 0.843) were comparable between the clinical trial and the registry cohorts (Table 3; Figure 2A). In addition, median OS and one-year survival rates were comparable between the clinical trial and registry cohorts for all subgroups analysed: (a) AML with MRC (AML-MRC); (b) NCCN poor-risk cytogenetics; (c) NCCN intermediate-risk cytogenetics; and (d) normal karyotype (Figure 2B–E). Regarding response, achievement of red blood cell- (RBC-) and PLT-transfusion independence (TI) was also similar between trial and registry cohorts, respectively (*p* = 0.7522, *p* = 1.0000; Table 3). However, the overall response rate (ORR; CR/CRi/partial response (PR)) was significantly higher in the AZA-AML-001 trial, as compared to the AAR (30.4% vs. 18.9%, *p* = 0.0379), which was due to a lower rate of CR/CRi in the registry population.

Univariate and multivariate Cox regression analyses of OS were performed to evaluate the similarity of the two groups adjusted for baseline covariates (Table 4). The baseline variables age as a continuous variable (hazards ratio (HR): 1.02; 95% confidence interval (CI): 1.00, 1.04, *p* = 0.0182), age < versus ≥75 years (HR: 0.70; 95% CI: 0.54, 0.90, *p* = 0.0053), PLT-TD (HR: 0.68; 95% CI: 0.53, 0.88, *p* = 0.0028), ECOG-PS (HR: 0.54; 95% CI: 0.41, 0.71, *p* < 0.001) and NCCN cytogenetic risk (HR: 0.51; 95% CI: 0.39, 0.66, *p* < 0.001) had a significant impact on survival in univariate analysis (Table 4). Of note, patient affiliation with the clinical trial or the registry cohort did not have an impact on OS (HR: 1.02; 95% CI: 0.78, 1.32, *p* = 0.8998).

In multivariate analysis, PLT-TD (HR: 0.69; 95% CI: 0.53, 0.90, *p* = 0.0057), ECOG-PS (HR: 0.65; 95% CI: 0.48, 0.87, *p* = 0.0041) and NCCN cytogenetic status (HR: 0.51; 95% CI: 0.39, 0.67, *p* < 0.001) remained significant. As the baseline covariate “AML-001” versus “AAR (001-like)” was the variable most critical to this publication, this variable was kept in the final multivariate model, and the two groups were comparable even after adjustment for significant baseline covariates (HR: 1.11; 95% CI: 0.84, 1.47, *p* = 0.4509), indicating no relevant difference between the outcomes of patients from either cohort (Table 4).

Having determined no relevant differences in baseline variables, treatment-related characteristics or various measures of outcome between the AZA-AML-001 trial and the “real-world” cohorts of the AAR, both cohorts were pooled (*n* = 309) for outcome analyses: median OS was 10.3 (95% CI: 8.65, 12.56) months, and the Kaplan–Meier (KM) estimate of one-year survival rate was 45.8%.

### 2.2. Registry Subsets Meeting Clinical Trial Inclusion Criteria versus All WHO-AML Patients

Baseline characteristics were similar between the registry subset meeting clinical trial inclusion criteria (“AAR (001 like)”) and all WHO-AML patients included within the registry (“AAR (WHO-AML)”) treated with azacitidine front-line, with the exception of BM blast count, which was expectedly lower for the “WHO-AML” patient subgroup as these also included patients with 20%–30% BM blasts (Table 1). Treatment-related characteristics were similar between the two groups (Table 2). The occurrence of Grade 3–4 treatment-emergent adverse events (TEAEs) per total applied azacitidine cycle was similar for the “001-like” and “WHO-AML” subsets (0.24 vs. 0.22; Table S1). In all WHO-AML patients treated with azacitidine front-line within the AAR, 35% of all TEAEs and 20% of all Grade 3–4 TEAEs were deemed as azacitidine-related; 9% of Grade 3–4 azacitidine-related AEs resulted in hospitalization, 6% in dose interruption, 9% in dose reduction and 3% in termination of treatment with azacitidine (Table S1).

**Figure 3:**
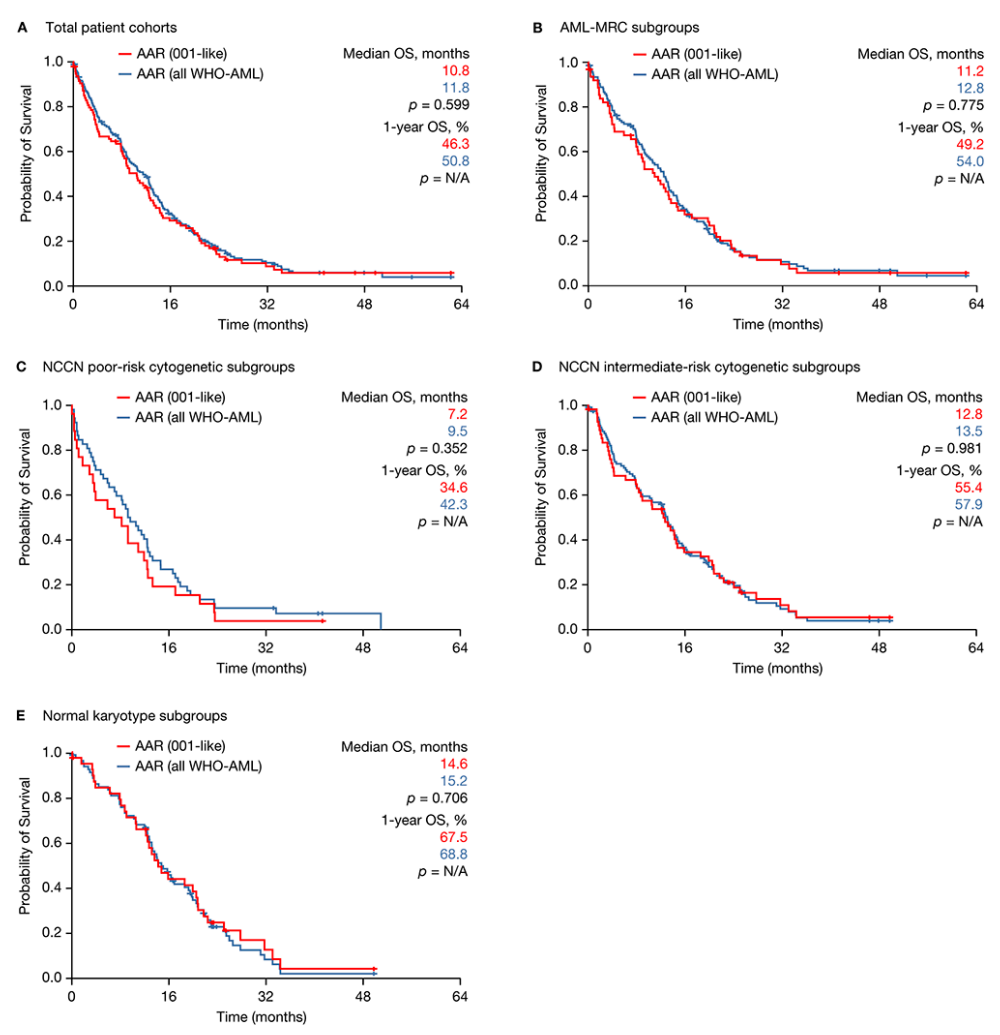
Median OS and one-year survival rates in AML patients with >30% BM blasts and <15 G/L WBC and WHO-AML patients treated with azacitidine front-line within the Austrian Azacitidine Registry. (**A**) Total patient cohorts; (**B**) AML-MRC; (**C**) AML with NCCN poor-risk cytogenetics; (**D**) AML with NCCN intermediate-risk cytogenetics; and (**E**) Normal karyotype.

Response, including ORR, TI and median CR/CRi duration, was similar between the “001-like” and “WHO-AML” subsets (Table 3). Other outcome measures including one-year survival rate and 30-day mortality rate, as well as median OS, median RFS duration and median EFS duration were also similar (Table 3; Figure 3A). The same held true in the subgroups of patients with AML-MRC, NCCN poor-risk cytogenetics, NCCN intermediate-risk cytogenetics and normal karyotype (Table 3; Figure 3B–E).

## 3. Discussion

Currently, both physicians and regulatory agencies are placing a stronger focus of attention on the performance of new drugs in the routine clinical practice setting, rather than merely relying on results obtained in clinical trials, and pressure is rising to recapitulate such results in unselected patients in clinical practice [22,23,29,30,31,32,33,34].

The EMA has reacted to this pressure by publishing its intention to “expand the use of patient registries” [34].

In anticipation of a need to test and potentially confirm the efficacy of azacitidine in a real-world population, the AAR established a platform to document the off-label use of azacitidine even before azacitidine was approved for the treatment of AML in the European Union and before the AZA-AML-001 trial was initiated. This indicates the lack of alternative treatments, an unmatched medical need and the high expectation of physicians that azacitidine would provide clinical benefit for their patients. Physicians’ anticipation and conviction that they were acting in the best interest of their patients, independent of the current EMA label, were based on previously published results in patients with higher-risk MDS and AML [7,8,9,10]. The AAR of the AGMT Study Group is a national registry with Ethics Committee approval, which collects data on MDS, CMML and AML patients specifically treated with hypomethylating agents. Data entry into a study-specific eCRF is performed by clinical trial personnel and/or the treating physicians, after the collection of patient written informed consent from all patients alive at the time of data entry. In contrast, other study groups have collected data from all patients diagnosed with MDS/AML [24] within registries or compassionate use programs approved by internal review boards [35], with informed patient consent. Some have also used anonymised minimal datasets of the entire country population obtained via medical claims from healthcare insurance companies and/or national cancer and/or leukaemia registries to assess treatment of patients with MDS and/or AML [5,36,37,38].

In a collaboration effort with the sponsors, data owners and authors of the AZA-AML-001 trial, this study compared data of AML patients treated with azacitidine front-line within the setting of the AZA-AML-001 clinical trial [21] (and subsets thereof [39,40,41]) versus the clinical practice registry setting of the AAR. To our best knowledge, such a comparison of patient-level data between a randomised phase 3 clinical trial and a nationwide registry has not been published in AML before.

For the first analysis, patients were selected according to the most relevant inclusion criteria of the AZA-AML-001 trial, namely the presence of WHO-AML, front-line treatment with azacitidine, BM blasts >30% and a WBC count of ≤15 G/L (Figure 1). Other clinical trial inclusion/exclusion criteria were not used in order to maintain the additional information gained by a “less-controlled, wider-ranging, naturalistic (registry) setting” [32]. Baseline and treatment characteristics, as well as safety and survival data were similar between the two groups, despite the fact that the ORR, mainly due to higher CR/CRi rates, was significantly higher in the clinical trial than in the registry setting (Table 3). While achievement of CR/CRi remains the primary treatment goal for all AML patients irrespective of age, we and others have previously shown that achievement of CR/CRi is not necessarily a prerequisite for OS benefit in AML patients treated with non-intensive therapeutic options [42,43,44,45,46]. Interestingly, a recent analysis of a Danish population-based cohort reports that AML patients treated with intensive chemotherapeutic regimens within clinical trials not only had higher CR rates compared with patients treated off-trial (80.2% vs. 68.5%), but also had superior one-year survival (61% vs. 45% for patients older than 60 years). A possible explanation for the worse survival observed in AML patients treated with standard intensive therapy regimens off-trial in Denmark might be that they had a less favourable profile than patients treated on-trial [26].

We thus confirm the safety and efficacy of front-line azacitidine in these patients, as well as for all subgroups analysed. This included stratification according to cytogenetic risk category and AML-MRC. These results also indicate that there was no bias towards improved patient outcome within the AZA-AML-001 trial through the use of more stringent exclusion criteria as compared to the registry population. Pooled data from the clinical trial and the registry cohorts revealed a median OS of 10.3 months and a one-year survival rate of 45.8% for 309 AML patients. We thus expand on recently published data from the AZA-AML-001 trial [21] and confirm the efficacy of azacitidine in this patient group.

We further aimed to evaluate whether the efficacy of azacitidine might be limited to patient subsets fulfilling the AZA-AML-001 clinical trial inclusion criteria (i.e., front-line treatment with azacitidine, BM blasts >30% and a WBC count of ≤15 G/L). In this regard, no relevant difference in baseline parameters, treatment-related parameters, TEAEs, response and survival outcomes existed between patients of the AAR that met clinical trial inclusion criteria versus all WHO-AML patients (Table 1, Table 2 and Table 3; Table S1; Figure 3A). These results further confirm that the inclusion criteria used by the AZA-AML-001 trial did not select for a patient population with particularly good or particularly bad features and/or treatment outcomes. These data expand on the clinical trial data by demonstrating the safety and efficacy of front-line azacitidine in all patients with WHO-AML, irrespective of BM blast or WBC counts. This remained true when analysing patient subgroups stratified according to NCCN cytogenetic risk status and the presence of MRC separately (Figure 3B–E). In this regard, it seems noteworthy that azacitidine may overcome baseline factors generally considered to be indicators of adverse prognosis in AML, such as adverse cytogenetics [46], elevated WBC count [47] and MRC [48]. For example, azacitidine showed significant survival benefit over conventional care regimens in the NCCN poor-risk cytogenetic subgroup analysis of the AZA-AML-001 trial (median OS 6.4 vs. 3.2 months, *p* = 0.019; one-year survival rate 30.9% vs. 14.0%) [39], and we have previously reported that neither WBC < versus ≥15 G/L (12.8 vs. 13.5 months, *p* = 0.250) [14], nor the presence of MRC adversely affect OS of AML patients treated with azacitidine front-line (13.2 vs. 8.9 months, *p* = 0.104) [13].

It is clear that observational studies cannot replace clinical trials, but they do play a well-accepted complementary role. One might argue that the retrospective, uncontrolled nature of a registry setting is a drawback of the current analyses. However, precisely this fact represents one of the acknowledged advantages of registry settings. The necessity to assess the effect of new drugs in patients that often escape inclusion in clinical trials, in order to gain additional insights into a drug’s ability to achieve its intended use, safety and outcome in the routine clinical practice setting, in contrast to the confined and controlled environment of a clinical trial, is supported by several groups, including the EMA [22,23,29,30,31,32,33,34,37,49,50,51].

Another recurrent concern about registries is the potential presence of a bias regarding the inclusion of patients. In the AAR, 20% of all patients (and 40% of patients aged ≥70 years) diagnosed with AML across Austria were included in the registry between 2008 and 2012 (Table S2). In comparison, a large retrospective analysis of 4416 patients with MDS from the SEER database revealed that only 7.7% of patients diagnosed before May 2007 and 13.4% of patients diagnosed thereafter were treated with hypomethylating agents in the U.S. [52]. Similarly, a more recent and larger analysis (*n* = 8580) from the Medicare database (cut-off date: 31 December 2012) published similar findings, namely that only 14.7% of MDS patients aged ≥66 years had received hypomethylating agents [53]. The Medicare database used the International Classification of Diseases for Oncology 3rd edition (ICD-O-3), which included patients with AML and 20%–30% BM blasts. No data on the use of hypomethylating agents has been published for all types of WHO-AML, including AML with >30% BM blasts. In comparison, seven AML patients were randomised in the U.S. during the recruitment period of 30 months (2.5 years) in the international AZA-AML-001 trial, which accounts for approximately 0.06% of all newly-diagnosed AML patients in the U.S. during this time period (4.889 new AML cases per year in 2013 [54]). In light of all of the above, an inclusion rate of 20% of AML patients of all ages and 40% of AML patients >70 years in the AAR (cut-off date: 31 December 2012) seems to be a very high nationwide coverage of AML patients treated with azacitidine, indicating limited selection bias. In addition, 20% of patients had received only one cycle of azacitidine, 64% ≤3 treatment cycles, 26% ≥3 comorbidities and 26% ECOG-PS ≥2, further indicating limited selection bias.

## 4. Materials and Methods

### 4.1. Design and Aim of the Study

This analysis selected patients with AML receiving azacitidine as front-line therapy within the AZA-AML-001 trial and the AAR. In total, 429 patients were identified that met this criterion, 241 from the AZA-AML-001 trial and 193 from the AAR (Figure 1). In the first analyses, we further selected for the presence of >30% BM blasts and ≤15 G/L WBC and directly compared baseline and treatment characteristics, as well as treatment outcomes and OS of patients included within the AZA-AML-001 trial (*n* = 214; “AML-001” subset) versus patients included within the AAR (*n* = 95; “AAR (001-like)” subset), using patient-level data from both cohorts (Figure 1). Comparative analyses of patient subsets from the AZA-AML-001 trial and the AAR grouped according to NCCN cytogenetic risk categories, as well as the presence of MRC were also performed.

Secondly, we wanted to explore whether the strict inclusion criteria of the AZA-AML-001 trial hampered the generalizability of the outcome data, as compared to the registry population. We next pooled patient data from the “AML-001” subset and the “AAR (001-like)” subset (*n* = 309), for outcome analyses (Figure 1).

Thirdly, we further aimed to evaluate whether there was a difference in baseline factors, treatment-related factors, TEAEs and the efficacy of azacitidine as front-line therapy for AML patients of the AAR who met clinical trial inclusion criteria (“AAR (001-like)” subset) versus all comers (“AAR (WHO-AML)” subset) (Figure 1).

### 4.2. Setting of the Study

The multicentre, randomised, open-label, parallel-group AZA-AML-001 trial (NCT01074047; registered February 2010) evaluated the efficacy and safety of azacitidine versus conventional care regimens in 488 patients aged ≥65 years with newly diagnosed AML with >30% BM blasts and ≤15 G/L WBC and was conducted in 18 countries. Details regarding the study design and the randomization process have recently been published [21] (study start date June 2010; last patient randomised 5 November 2012; final data collection date 22 January 2014). The AAR (NCT01595295; registered May 2012) is a multicentre database that includes 900 patients with AML, MDS or chronic myelomonocytic leukaemia (CMML) who were treated with azacitidine during the course of their disease. The AAR was initiated by the AGMT Study Group in 2009 to provide representative insight into the clinical management of patients treated with azacitidine in a clinical practice setting in Austria [11,12,13,14,15,16,17,18,19,20,55,56]. The AAR adheres to published quality guidelines of the U.S. Department of Health and Human Services Agency for Healthcare Research and Quality (AHRQ) [57]. The final data collection date for this analysis was 31 May 2016.

### 4.3. Definitions and Endpoints

For all patients included in these analyses (*n* = 407), AML was defined according to WHO 2008 criteria [58]. Front-line therapy with azacitidine was defined as the absence of prior disease-modifying therapy (in the AZA-AML-001 trial, hydroxyurea was allowed up to 2 weeks before the screening haematology sample was taken). Cytogenetic risk was classified according to NCCN 2009 cytogenetic risk categories [59].

Median OS was defined as time from Day 1 (date of randomization for the AML-001 group; Day 1 of Cycle 1 azacitidine treatment for the AAR group) to death from any cause. Event-free survival (EFS; for all patients) and relapse-free survival (RFS; for patients achieving CR/CRi) were assessed from Day 1 to the occurrence of an event (events included treatment failure, progressive disease, relapse after CR/CRi, death from any cause or lost to follow-up, whichever occurred first). Participants who were still alive without any of these events were censored at the date of their last assessment.

Response was determined based on International Working Group (IWG) Response Criteria for AML [60]. Morphologic CR was defined as <5% blasts in the BM (histology and/or aspirate with marrow spicules (≥200 nucleated cells, absence of blasts with Auer rods) and an ANC of ≥1.0 G/L, a PLT count ≥100 G/L in the peripheral blood, as well as TI (no transfusions for 1–4 weeks prior to each assessment)). Morphologic CRi was defined as BM blasts <5% in histology and/or cytology, not meeting ≤1 of the peripheral blood CR criteria. PR was defined as an at least 50% reduction of baseline BM blast count in histology and/or aspirate (BM blasts must be >5% and <25%) and an ANC of ≥1.0 G/L, a PLT count ≥ 100 G/L in the peripheral blood, as well as TI (no transfusions for 1–4 weeks prior to each assessment)). No duration of these findings was required for confirmation of this response. The ORR included CR, CRi and PR [60]. RBC- and PLT-TI was defined as achievement of TI for at least 56 consecutive days and was assessed for patients who were transfusion dependent at baseline (≥1 transfusion within the 56 days prior to Day 1). Duration of CR/CRi was defined as the time from the date CR/CRi was first documented until the date of documented relapse/disease progression from CR/CRi. Participants who were lost to follow-up without documented relapse or were alive at last follow-up without documented relapse were censored at the date of their last response assessment.

AEs were assessed according to the Common Terminology Criteria for Adverse Events (CTCAEv.4; [61]). The safety population included all patients who had received at least one dose of azacitidine and had at least one safety assessment thereafter. TEAEs were defined as new or worsening AEs between the time of first azacitidine dose and the end of the safety follow-up period, which was set at 28 days after the last dose of azacitidine. Treatment-emergent haematological toxicity was calculated from differential blood counts and transfusion status at Day 1 of each respective azacitidine treatment cycle.

### 4.4. Characteristics of Participants

Inclusion criteria of the AZA-AML-001 trial comprised newly-diagnosed patients with AML, ineligibility for HSCT, age ≥65 years, BM blasts >30%, WBC ≤15 G/L, ECOG-PS ≤2 and intermediate- or poor-risk cytogenetics (NCCN 2009 criteria); exclusion criteria included antecedent chronic CMML or other myeloproliferative neoplasm (MPN) and concurrent malignancies [21]. The only inclusion criteria of the AAR were a diagnosis of AML, MDS or CMML and treatment with at least one dose of azacitidine. No formal exclusion criteria existed, as the aim was to document the use and efficacy of azacitidine irrespective of age, comorbidities and/or previous lines of treatment. Further details have recently been published in this journal [8,13].

### 4.5. Processes, Interventions and Comparisons

This is a non-interventional retrospective comparative analysis of patient-level data of the above-defined patient populations with the above-defined aims and using the below-defined methods.

### 4.6. Statistical Analyses

Patient-level data for both studies were analysed by the sponsor of the AZA-AML-001 trial (in contrast to recently-presented data of an indirect comparison [15] based on previously-reported results [21,39,40]). No patients were excluded from efficacy and safety analyses. Survival distribution functions were estimated by the KM method, and survival curves were compared using the log-rank method. Univariate and multivariate analyses using Cox-regression of OS adjusted for significant baseline covariates were performed. Covariates in the univariate analysis with a *p*-value < 0.10 were included in the multivariate model. A backward selection method with a stay-level of 0.1 was used as the criterion to select covariates for the final model. The study covariate (i.e., AML-001 vs. AAR) was kept in the final multivariate model.

## 5. Conclusions

We tested and confirmed the efficacy of azacitidine as a front-line agent in patients with AML treated within the AGMT-AAR. Data obtained within the AZA-AML-001 trial could be recapitulated both in an AAR cohort of patients meeting AZA-AML-001 trial inclusion criteria (AML, >30% BM blasts and WBC ≤15 G/L), as well as in a wider AAR patient population that included all patients with WHO-AML. We thus not only confirm the value of azacitidine as reported by the clinical trial in a real-world scenario, but also expand on these data by showing that the safety and efficacy of azacitidine as a front-line agent is not limited to patients meeting stringent clinical trial inclusion criteria. Instead, azacitidine appears efficacious as a front-line treatment in a wider patient population that includes all patients with WHO-AML.

## Figures and Tables

**Table 1:** Baseline characteristics of acute myeloid leukaemia (AML) patients treated with azacitidine front-line per patient subset.

Baseline Characteristics	AML-001 Trial	AAR (001-Like)	AAR (WHO-AML)
	Subset	Subset	Subset
	(*n* = 214)	(*n* = 95)	(*n* = 193)
Age, median (mean) [SD], years	76 (75.5) [5.6]	77 (75.2) [11.5]	77 (75.6) [10.2]
Age ≥75 years, %	58.4	56.8	58.5
Male, %	57.5	54.7	58.6
ECOG-PS, %			
0–1	76.6	67.3	67.9
2	23.4	23.2	24.4
3–4 ^a^	0	9.5	1.6
AML classification, %			
AML-MRC ^b^	57.0	66.3	70.8
AML-NOS	37.3	24.2	18.8
AML-RCA	2.3	4.2	3.7
t-AML	3.3	5.3	6.8
Antecedent haematological disease, %	19.6	25.3	31.6
Prior MDS, %	19.6	21.1	24.9
NCCN cytogenetic risk, %			
Good ^c^	0	2.1	2.4
Intermediate	65.0	58.9	66.9
Normal	47.7	42.1	47.3
Poor	34.6	27.4	30.8
BM blasts, median (range), %	73.0 (31–100)	55.5 (31–96)	40.0 (20–100)
BM blasts ≥50%, %	78.0	58.9	38.9
Number of cytopenias, %			
0–1	13.1	14.7	16.6
2–3	86.9	85.3	83.4
RBC-TD, %	70.1	60.0	52.9
PLT-TD, %	40.2	30.5	24.9
WBC, median (range), G/L	3.0 (0.3–14.7)	2.1 (0.6–14.4)	2.5 (0.6–74.1)
Hb, median (range), g/dL	9.5 (5.0–13.4)	9.1 (5.8–13.6)	9.1 (5.8–14.2)
ANC, median (range), G/L	0.3 (0–5.3)	0.5 (0–7.7)	0.6 (0–37.2)
PLT, median (range), G/L	54.0 (3–585)	49.0 (7–1.270)	52.0 (6–1,270)

^a^ ECOG-PS > 2 was an exclusion criterion in the AZA-AML-001 trial. Nine patients included within the AAR had an ECOG-PS > 2; ^b^ The definition of MRC in the AZA-AML-001 trial was based on the presence of NCCN poor-risk cytogenetics. This means that the following cytogenetic aberrations (included in the WHO definition of myelodysplasia-related changes) were not accounted for: -9q, -12p, -13q, -13, t(12p), t(2;11), t(3;5), t(3;21), t(5;7), t(5;10), t(5;17), t(11;16), isochromosome(17q), idic(X)(q13), possibly resulting in a lower number of patients within this subgroup. In addition, the presence of prior chronic myelomonocytic leukaemia and prior myeloproliferative neoplasia was an exclusion criterion in the AML-001 trial, possibly resulting in a slightly lower number of patients within this subgroup; and ^c^ NCCN cytogenetic good risk was an exclusion criterion in the AZA-AML-001 trial. Two patients were included within the AAR had NCCN good risk cytogenetics.

**Table 2:** Treatment characteristics of AML patients treated with azacitidine front-line per patient subset.

Treatment Characteristics	AML-001	AAR	AAR
	Trial	(001-Like)	(WHO-AML)
	(*n* = 214)	(*n* = 95)	(*n* = 193)
AZA cycles, median, *n*	6	5	6
(Mean) [SD]	(8.4) [7.1]	(8.5) [9.1]	(8.4) [6.0]
AZA cycles ≥6, %	50.0	46.3	51.3
AZA cycles ≥12, %	28.5	24.2	24.9
Days of AZA application, median, days	42	34	39
(Mean) [SD]	(58.0) [49.8]	(55.8) [61.1]	(57.1) [57.3]
Daily of AZA dose, median, mg	130.1	131.6	132.0
(Mean) [SD]	(129.4) [17.8]	(128.7) [26.5]	(126.4) [33.3]
Reasons for AZA discontinuation, %			
AE/no response/relapse/PD/death	66.8	74.8	73.1
Withdrew consent/patient’s wish	11.7	9.5	7.3
Others	11.7	9.5	11.4
Still on AZA at study closure	9.8	6.3	8.3

**Table 3:** Outcome of AML patients treated with azacitidine front-line per patient subset.

Outcome	AML-001 Trial (*n* = 214)	AAR (001-Like) (*n* = 95)	*p*-Value	AAR (001-Like) (*n* = 95)	AAR (WHO-AML) (*n* = 193)	*p*-Value
Median OS, mo	9.9	10.7	0.9553 ^a^	10.7	11.8	0.599 ^a^
Median RFS (CR/CRi), mo	16.3	13.8	0.6817 ^a^	13.8	13.3	0.621 ^a^
Median EFS (all pts), mo	6.9	8.3	0.2909 ^a^	8.3	8.1	0.941 ^a^
Median CR/CRi duration, mo	8.6	11.1	0.1740 ^a^	11.1	11.5	0.818 ^a^
1-Year survival, %	54.2	53.7	0.843 ^b^	53.7	50.8	0.476 ^b^
30-Day mortality, %	7.0	8.4	0.924 ^b^	8.4	7.8	0.848 ^b^
ORR (CR, CRi, PR), %	30.4	18.9	0.0379 ^b^	18.9	23.1	0.685 ^b^
RBC-TI, %	39.3	42.1	0.7522 ^b^	42.1	42.2	0.517 ^b^
PLT-TI, %	37.2	35.7	1.0000 ^b^	35.7	41.7	0.688 ^b^

^a^ Median times for OS, RFS, EFS and CR/CRi duration were estimated by the KM method, and the *p*-value was based on the log-rank test; and ^b^ Calculated according to the χ-squared test for categorical variables and the *t*-test for continuous variable.

**Table 4:** Univariate and multivariate analysis of the effects of baseline covariates on the OS of AML patients treated with azacitidine front-line with >30% BM blasts and <15 G/L WBC within the AZA-AML-001 trial and the AAR.

**Univariate Analysis**		
Baseline Parameter	HR (95% CI)	*p*-Value
Study group (AML-001 vs. AAR)	1.02 (0.78, 1.32)	0.8998
Age (as a continuous variable)	1.02 (1.00, 1.04)	0.0182
Age (<75 vs. ≥75 years)	0.70 (0.54, 0.90)	0.0053
Gender (female vs. male)	0.82 (0.64, 1.05)	0.1243
RBC-TD (No vs. Yes)	0.89 (0.69, 1.16)	0.3857
PLT-TD (No vs. Yes)	0.68 (0.53, 0.88)	0.0028
ECOG-PS (0–1 vs. ≥2)	0.54 (0.41, 0.71)	<0.001
MDS-related changes present (Yes vs. No)	0.90 (0.70, 1.16)	0.4326
Prior MDS (No vs. Yes)	1.01 (0.74, 1.38)	0.9366
No. of cytopenias at baseline (0–1 vs. 2–3)	0.83 (0.58, 1.19)	0.3082
NCCN cytogenetic risk (Intermediate vs. Poor)	0.51 (0.39, 0.66)	<0.001
BM blasts (30%–49% vs. ≥50%)	0.90 (0.69, 1.18)	0.4511
WBC (as a continuous variable)	1.00 (0.97, 1.04)	0.8400
ANC (as a continuous variable)	1.05 (0.93, 1.17)	0.4514
PLT count (as a continuous variable)	1.00 (1.00, 1.00)	0.1487
Hb (as a continuous variable)	0.96 (0.88, 1.05)	0.3344
**Multivariate Analysis**		
Baseline Covariate	HR (95% CI)	*p*-Value
Age (<75 vs. ≥75 years)	0.76 (0.58, 0.98)	0.0366
PLT-TD (No vs. Yes)	0.69 (0.53, 0.90)	0.0057
ECOG-PS (0–1 vs. ≥2)	0.65 (0.48, 0.87)	0.0041
NCCN cytogenetic risk (Intermediate vs. Poor)	0.51 (0.39, 0.67)	<0.001
AML-001 vs. AAR ^a^	1.11 (0.84, 1.47)	0.4509

**^a^** As this covariate was the variable most critical to the intended analysis, it was kept in the final multivariate analysis model.

## Supplementary Materials

Supplementary materials can be found at www.mdpi.com/1422-0067/18/2/415/s1.

## Abbreviations

AAR	Austrian Azacitidine Registry
AE	Adverse event
AGMT	Arbeitsgemeinschaft Medikamentöse Tumortherapie
AHRQ	Agency for Healthcare Research and Quality
AML	Acute myeloid leukaemia
ANC	Absolute neutrophil count
AZA	Azacitidine
BM	Bone marrow
BSC	Best supportive care
CI	Confidence interval
CMML	Chronic myelomonocytic leukaemia
CR	Complete response
CRi	Morphologic CR with incomplete blood count recovery
ECOG-PS	Eastern Cooperative Oncology Group Performance Status
eCRF	Electronic case report form
EFS	Event-free survival
EMA	European Medicines Agency
HSCT	Hematopoietic stem cell transplantation
Hb	Haemoglobin
HR	Hazards ratio
IWG	International Working Group
KM	Kaplan–Meier
MDS	Myelodysplastic syndromes
MPN	Myeloproliferative neoplasm
MRC	Myelodysplasia-related changes
NCCN	National Comprehensive Cancer Network
NOS	Not otherwise specified
ORR	Overall response rate
OS	Overall survival
PLT	Platelet
PR	Partial response
pts	Patients
RBC	Red blood cell
RCA	Recurrent cytogenetic abnormalities
RFS	Relapse-free survival
SD	Standard deviation
t-AML	Therapy-related AML
TD	Transfusion dependence
TEAE	Treatment-emergent adverse event
TI	Transfusion independence
vs.	Versus
WBC	White blood cell
WHO	World Health Organisation
