# Supplementary material for: Azacitidine for Front-Line Therapy of Patients with AML: Reproducible Efficacy Established by Direct Comparison of International Phase 3 Trial Data with Registry Data from the Austrian Azacitidine Registry of the AGMT Study Group
Source: Int J Mol Sci. 2017 Feb 15;18(2):415. doi: 10.3390/ijms18020415 (PMC5343949; doi:10.3390/ijms18020415)
Supplement: Supplementary file 1 [file ijms-18-00415-s001.pdf]

Supplementary Materials: Azacitidine for Front-Line Therapy of Patients with AML: Reproducible Efficacy Established by Direct Comparison of International Phase 3 Trial Data with Registry Data from the Austrian Azacitidine Registry of the AGMT Study Group

Lisa Pleyer, Hartmut Döhner, Hervé Dombret, John F. Seymour, Andre C. Schuh, CL Beach, Arlene S. Swern, Sonja Burgstaller, Reinhard Stauder, Michael Girschikofsky, Heinz Sill, Konstantin Schlick, Josef Thaler, Britta Halter, Sigrid Machherndl Spandl, Armin Zebisch, Angelika Pichler, Michael Pfeilstöcker, Eva M. Autzinger, Alois Lang, Klaus Geissler, Daniela Voskova, Wolfgang R. Sperr, Sabine Hojas, Inga M. Rogulj, Johannes Andel and Richard Greil

Table S1. Treatment-emergent adverse events of AML patients treated with azacitidine front-line within the AAR.

**Treatment Emergent Adverse Events (TEAEs)**	**AAR (001-Like) Subset (*n* = 95)**	**AAR (WHO-AML) Subset (*n* = 193)**
TEAEs any grade, *n* total events/*n* total cycles	482/805 (0.60)	963/1699 (0.57)
TEAEs G3-4	195/805 (0.24)	376/1699 (0.22)
AZA-related any grade	69/805 (0.09)	193/1699 (0.11)
AZA-related G3-4	37/805 (0.05)	79/1699 (0.05)
TEAEs any grade (G3-4), % pts	89.5 (77.9)	89.6 (76.7)
AZA interruption	27.4	32.6
AZA dose reduction	23.2	20.2
AZA termination	37.9	36.8
Hospitalization	52.6	53.9
AZA-related TEAEs any grade (G3-4), % pts	29.5 (17.9)	34.7 (20.2)
AZA interruption	4.2	6.2
AZA dose reduction	12.6	8.8
AZA termination	3.2	2.6
Hospitalization	8.4	8.8
TE thrombocytopenia G3-4, % pts	42.1	41.5
TE neutropenia G3-4, % pts	31.6	35.2
TE anaemia G3-4, % pts	47.4	49.2
Infections any grade, % pts	72.6	69.9
Infections (G3-4)	38.9	21.8
Pulmonary infection (any grade), % pts	36.8	30.6
Febrile neutropenia, % pts	24.2	22.3
Sepsis, % pts	9.5	9.8
Nausea (any grade), % pts	10.5	10.4

AAR, Austrian Azacitidine Registry; AML, acute myeloid leukaemia; AZA, azacitidine; TEAE, treatment-emergent adverse event; WHO, World Health Organization.

Table S2. Number of AML diagnoses per year in Austria and patient recruitment to the AAR.

Year	AML diagnoses in Austria [a,b]		AZA Start in AAR [c]		
	All Adults	≥70 Years	*n* pts	% of Adults with AML	% of pts ≥70 Years
2007	318	140	10	3	7
2008	279	146	22	6	15
2009	269	132	55	20	15
2010	316	159	59	19	37
2011	266	154	64	24	42
2012	324	159	78	24	49
Total	1448	731	288	20	39

Incidence of new diagnoses of AML (diagnosis C92.0), as well as the numbers of AML patients included within the AAR and the number of centres including patients in the registry per respective year (note: as Ethics Committee approval was obtained on 1 February 2009, data entry commenced as of this time point). The respective numbers for the centre of Salzburg only are also shown. The use of AZA in AML was not common prior to approval by the EMA and has increased in use with maturing experience with the drug. Data on treatment of AML patients were not available from Statistics Austria. It was not our aim to report on all AML patients in Austria, but on the majority of these patients that received AZA at any time point during the course of their disease. [a] Data received from Statistics Austria on 7 March 2016; no update for the years 2013–2015 was available on 7 March 2016; [b] Please note that the patients started on AZA in a respective year are not necessarily those diagnosed in the same year; and [c] AAR, Austrian Azacitidine Registry; AML, acute myeloid leukaemia; AZA, azacitidine; EMA, European Medicines Agency; WHO, World Health Organization.
